# Intratesticular versus intraperitoneal Busulfan administration: a comparative study on spermatogenesis suppression in quails and chickens

**DOI:** 10.1016/j.psj.2024.103890

**Published:** 2024-05-23

**Authors:** Noor R. Wattad, Eden Ozer, Stefanie Altgilbers, Claudia Klein, Eyal Cohen, Ohad Zuckrman, Eitan Sessler, Tamar Hadad, Yehonatan Alcalay, Uri Abdu

**Affiliations:** ⁎ Department of Life Sciences, Ben-Gurion University of the Negev, Beer Sheva 84105, Israel; † COPIA Agro & Food, Herzliya, 4672501, Israel; ‡ Friedrich-Loeffler-Institut – Federal Research Institute for Animal Health (FLI), Institute of Farm Animal Genetics, Department of Biotechnology, Neustadt 31535, Germany; § BugEra, Insect Biotechnology Research, Beer Sheva 84105, Israel

**Keywords:** busulfan, chicken, quail, spermatogenesis, sterility, testis

## Abstract

Generation of transgenic birds can be achieved by temporal suppression of endogenous spermatogenesis in males prior to primordial germ cell implantation. One of many established methods to induce male sterility is the intraperitoneal injection of busulfan, an alkylating agent. Nevertheless, the use of busulfan injections, which may also affect hematopoietic stem cells, carries the risk of potential lethality in animals. Given their safety and non-toxic nature, it has been demonstrated that intratesticular busulfan injections in mammals are less effective than intraperitoneal injections. This study aimed to compare, for the first time, the sterility and toxicity effects of intraperitoneal vs. intratesticular busulfan injections in quail and chickens. Our experimental design involved a previously established single intraperitoneal busulfan injection of 40 mg/kg of body weight (**BW**). In quail, busulfan was then administered intratesticularly at 3 different concentrations (6, 12, and 20 mg/kg BW), while in chickens, the working concentration was 20 mg/kg BW. We found that a single intraperitoneal busulfan injection of 40 mg/kg of BW resulted in 100% mortality in the treated roosters. In quails, however, this concentration only caused a temporary suppression of fertility for a 15-d period. Moreover, we found that a higher dose of intratesticular injection of busulfan is required to suppress spermatogenesis in quail (20 mg/kg BW) compared to mammals (4 mg/kg BW). Following these findings, we further confirmed that intratesticular injection of 20 mg/kg BW busulfan into roosters did not affect their overall viability. However, it induced a temporary state of male sterility, consistent with the effects observed with intraperitoneal injections. Hence, our data demonstrate that quail and chicken respond differently to busulfan administration. Furthermore, the present study provides evidence that direct injection into the rooster testes causes less physiological stress than intraperitoneal injection.

## INTRODUCTION

In the biological sciences, avian species have historically served as model animals for the study of embryonic development, behavior, physiology, and neuroscience (Konishi et al., 1989). More specifically, transgenic birds are widely used as a convenient research platform (Love et al., 1994; Scott & Lois, 2005; Serralbo et al., 2020), and there are many reliable methods for generating transgenic birds (Sid & Schusser, 2018). The production of germline chimeric chicken can be achieved by in vivo or in vitro manipulation of primordial germ cells (**PGCs**). In the past, transgenic chickens have been generated through in vivo approaches, that is, injection of viral particles (Salter et al., 1987; McGrew et al., 2004), plasmid DNA (Love et al., 1994), and the application of Tol2 transposase (Tyack et al., 2013). The recently established genetic in vitro manipulation of circulating or gonad-derived PGCs of the chicken embryo with subsequent reintroduction into the embryonic vasculature (van de Lavoir et al., 2006) provided a new direction in generating transgenic chicken. However, a reliable method to establish a long-term culture of quail PGCs has not been reported to date. Thus, current approaches for generating transgenic quails in vivo are based on using either retroviral vectors (Mizuarai et al., 2001), lentiviral vectors (Scott and Lois, 2005), or the Tol2 transposase system (Serralbo et al., 2020).

*In vitro*-manipulated PGCs can be used to generate transgenic birds either by injection into the vascular system of developing embryos (van de Lavoir et al., 2006) or by intratesticular injection into adult males (Trefil et al., 2017). However, transplantation of genetically modified PGCs either into PGC-depleted embryos (Nakamura et al., 2008, 2010) or into the spermatogonial stem cells (**SSC**)-depleted testes of the recipient (Trefil et al., 2006; Ghadimi et al., 2017) might be more effective due to the elimination of the native PGCs or SSCs and, thus, reduced competition with the genetically modified PGCs. This approach is aimed at increasing the probability of producing a modified offspring (Nakamura et al., 2008, 2010; Trefil et al., 2017).

Two methods were developed to eliminate endogenous PGC's or SSC in chicken and quail recipients prior to cell transplantation. The first method is gamma irradiation, which has been used only in chickens (Trefil et al., 2006, 2017). The second method is the intraperitoneal injection of an alkylating agent, busulfan (1,4-Butanediol dimethanesulfonate), which has been used both in chickens (Yu et al., 2010; Tagirov & Golovan, 2012; Ghadimi et al., 2017) and in quails (Jones & Jackson, 1972; Kim et al., 2018). According to previous research (Ghadimi et al., 2017), gamma radiation and busulfan treatment have the same effect on sperm reduction in chickens. Additionally, histological examinations demonstrate that both treatments significantly reduce the diameter of seminiferous tubules and the thickness of epithelium in the testicles.

The efficiency of gamma irradiation depends on the dose of radiation and the age of the animal at the time of exposure (Park et al., 2010; Ghadimi et al., 2017). However, this course of treatment requires specialized facilities and equipment. Busulfan injections, on the other hand, are routinely used in human medicine to treat cancer by alkylating deoxyribonucleic acid (**DNA**), thus damaging the proliferating cells. Similarly, busulfan is frequently used for the depletion of endogenous PGCs in chicken embryos (Nakamura et al., 2008, 2010) or SSCs in quails (Kim et al., 2018) prior to the transplantation of genetically modified cells. To date, the precise molecular mechanism underlying busulfan's impact on spermatogenesis remains uncertain. It has been suggested that busulfan disrupts DNA structure, inhibiting the proliferation and differentiation of SSCs, and induces apoptosis. Furthermore, busulfan is implicated in the degradation of vimentin filaments and intercellular cell adhesion molecule-1 (**ICAM-1**), while also interfering with differentiation factors, thereby hindering the maturation of SSCs (Zhao et al., 2023). Nonetheless, this method is significantly more convenient compared to gamma irradiation; however, the cytotoxicity of busulfan (which is dose-dependent) can be lethal to the treated animals.

In research literature, intratesticular injections of busulfan were reported to specifically target spermatogenesis without adverse toxic effects at 4 mg/kg of body weight (**BW**) concentration in mice (Qin et al., 2016a,b), 2 to 3 mg/kg BW in pigs (Lin et al., 2017), 1 to 2 mg/kg BW in ram lambs (Rasouli et al., 2020), 5 mg/kg BW in rats (Mobarak et al., 2022), and 2 mg/kg BW in goats (Singh et al., 2022). However, there is no documentation regarding direct testicular injections of busulfan in avian models. Thus, this study aims to evaluate the impact of administering busulfan injections intratesticularly vs. intraperitoneally on the sterility and mortality rates of male quails and chickens, with the ultimate goal of refining the protocol for generating transgenic birds.

In this study, we demonstrated that busulfan injection, delivered either intraperitoneally or intratesticularly, had no effect on male viability but effectively induced male sterility in quails. In chickens, however, intraperitoneal injection of busulfan was lethal to males, while intratesticular injection did not affect the survival of the injected animal and resulted in temporary male sterility, as intended.

## MATERIALS AND METHODS

### Animal Care

Animal welfare and experimental procedures conformed to the Institutional Guidelines for the Care and Use of Laboratory Animals at Ben-Gurion University of the Negev (**BGU**), Israel. All efforts were made to minimize animal suffering and the number of animals used. White Leghorn chickens (*Gallus gallus domesticus*) and Japanese quails (*Coturnix japonica*) were maintained according to a standard management program at the university's Avian facility (BGU, Israel). All procedures, including chicken and quail maintenance, reproduction, and sample collection, were governed by standard operating protocols fully accredited by the Association for Assessment and Accreditation of Laboratory Animal Care International (**AAALAC**).

### Fertility Assay

Two-month-old male Japanese quails and 6-mo-old Lohmann roosters were kept in individual cages, along with three females each, under standard husbandry conditions: 21°C, 45 to 60% relative humidity. Health status, feed and water intake were monitored daily. Prior to busulfan injections, fertility of males and females were monitored for 2 wk by checking embryonic development after 50 to 55 h of incubation, where embryo development is clearly visible (Hamburger and Hamilton, 1951). In this study, fertility assessment involved analyzing the mean number of fertilized eggs, which presents a quantitative measure that summarizes the overall reproductive performance within the specified experimental parameters.

### Intratesticular and Intraperitoneal Injections

Busulfan (Sigma Aldrich, Rehovot, Israel, B2635) was dissolved in dimethyl sulfoxide (**DMSO**) (Fisher Bioreagents, MA) and injected using a 29G insulin syringe. In quails, 0.15 mL of busulfan was injected either intraperitoneally at a single dose of 40 mg/kg BW concentration or intratesticularly at 3 different concentrations (6, 12, and 20 mg/kg BW). In roosters, 0.5 mL of busulfan was administered either intraperitoneally at a single dose of 40 mg/kg BW concentration or intratesticularly at a 20 mg/kg BW concentration. Control groups received vehicle-only (i.e., DMSO) injections at volumes equivalent to the respective experimental groups. Prior to the intratesticular injections, quails and roosters were anesthetized by liquid isoflurane inhalation. Next, the animals were placed on one side of the body, and a 2 cm incision was made between the last two ribs of the thorax using sterile surgical blades (no. 11; Swann Morton) to expose the testicles for a direct busulfan injection. The wound was closed using an Assut suture (Nylon 4/0, 19mm, 3/8 CRC). The animals that underwent surgery were kept in individual cages for 5 to 7 d to allow recovery; then, they were placed in cages with three females each, and egg fertility was assessed as described above.

### Tissue Preparation and Sectioning

For histological examination, a total of nine quails were euthanized through CO_2_ asphyxiation upon concluding the experiment (i.e., three individuals at each of the 5, 10, and 15-day time points after the injection). The testes were extracted, weighed to assess the impact of busulfan on testis weight. Additionally, the gonado-somatic index, representing the relative weight of the testicles compared to the birds' overall body weight, was calculated and documented in Table 2. Following excision, the testicular tissue was dissected into 2mm cubical sections, and then immersed in 4% paraformaldehyde (Electron Microscopy Sciences, Hatfield, England) at 4°C overnight for fixation. Next, the tissue was dehydrated using increasing concentrations of EtOH (50% to 100%), followed by two steps of incubation with xylene, and then embedded in paraffin and molded into blocks. Tissue was cut into 5 μm sections using a Leica rotational microtome (Leica Microsystems, IL, USA).

### Immunohistochemistry Staining

Sections were mounted on StarFrost microscope slides and dried for 1 h on a hotplate heated to 45°C, then overnight at room temperature. Sections were deparaffinized using sequential xylene, 100% EtOH, 90% EtOH, 80% EtOH, H_2_O, and phosphate-buffered saline (**PBS**) washes. For the antigen-retrieval step, the sections were immersed in sodium citrate buffer (10 mM sodium citrate, 0.05% Tween 20, pH 6.0) or Tris-EDTA buffer (10 mM Tris Base, 1 mM EDTA Solution, 0.05% Tween 20, pH 9.0) and incubated in a heating bath at 95°C for 30 min. Next, the sections were incubated in a blocking solution (1% bovine serum albumin in PBS) for 1 h and washed with PBS prior to the staining. Sections were incubated with primary antibodies (rabbit monoclonal anti-DAZL (AB-ab215718) and rabbit polyclonal anti-Sox9 antibody (AB5535-25UG)) at 4°C overnight, followed by 1 h of incubation at room temperature with the secondary antibodies (Alexa Flour 488 goat Anti-Rabbit IgG (Invitrogen, MA) or Cy3 donkey Anti-Rabbit IgG (Jackson ImmunoResearch, Philadelphia, PA)). Hoechst dye was added to stain the nuclear DNA. Finally, all sections were washed with PBS and covered with coverslips for microscopic observation (Menzel Gläser); the slides were sealed with transparent nail polish. The sections were imaged using the ZEISS LSM 800 laser confocal scanning microscope.

### Statistical Analysis

Data was analyzed using a one-way ANOVA, followed by a post-hoc Tukey's test, and the analysis was conducted using SYSTAT 12 software. Differences were considered statistically significant at a *p*-value < 0.05.

## RESULTS

### The Effect of Busulfan Intraperitoneal Injections on Viability and Fertility in Quails and Roosters

Previously, it has been demonstrated that a single intraperitoneal injection of busulfan at a dosage of 40 mg/kg BW results in male infertility without causing mortality in quails (Kim et al., 2018). In our study, 8-wk-old male quails (n = 4) were subjected to intraperitoneal injections of busulfan at a 40 mg/kg BW concentration; each male was housed subsequently with 3 females, and egg fertility was assessed daily.

**Figure 1:**
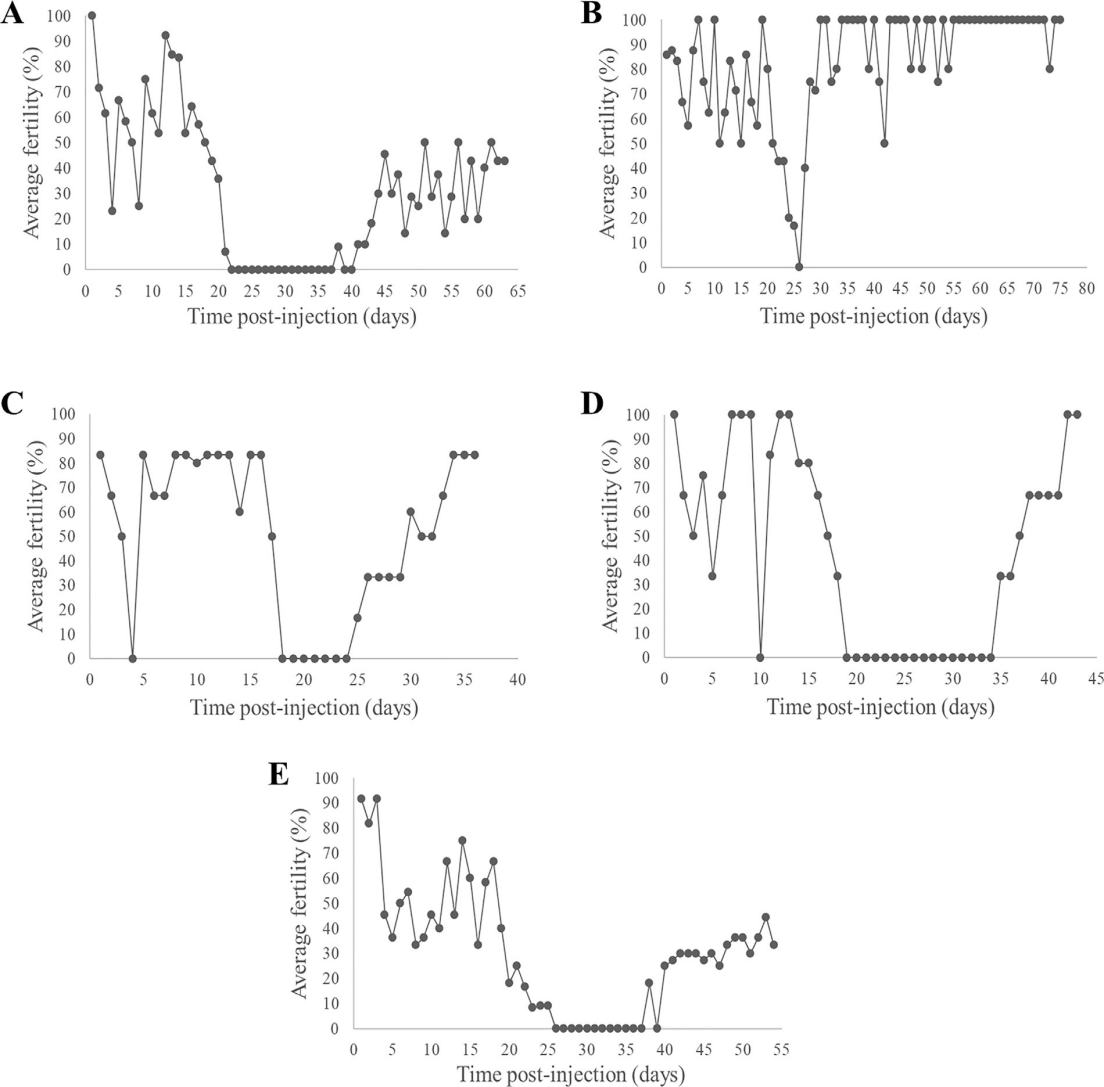
Effect of intraperitoneal and intratesticular busulfan injections on quail and chicken male fertility. (A) intraperitoneal injections of busulfan at 40 mg/kg in quail. (B) intratesticular injections of busulfan at 6 mg/kg in quail. (C) intratesticular injections of busulfan at 12 mg/kg in quail. (D) intratesticular injections of busulfan at 20 mg/kg in quail. (E) intratesticular injections of busulfan at 20 mg/kg in chicken.

First, our findings confirmed that a single intraperitoneal injection of busulfan at 40 mg/kg BW does not affect the survival of male quails. Second, evaluation of male fertility revealed a complete loss of fertility that occurred 20 ± 1.73 d after the injection (Table 1). The fertility restoration was observed on average after 41.3 ± 3.5 d postinjection, indicating the presence of a sterility window lasting 21.3 ± 3.79 d. Our analysis demonstrated that during the pre-sterility stage, encompassing the period from 1 d after the injection until the day before the fertility loss, approximately 58% ± 0.23 of the eggs were fertilized. Subsequently, none of the eggs were embryonated throughout the sterility window, spanning from the day of fertility loss until the day prior to fertility restoration. During the post-sterility stage, which extended from the day of fertility restoration until the end of the experiment, only 28% ± 0.15 of the eggs were fertile. The fertility of the male quails at the post-sterility stage was significantly lower compared to the pre-sterility stage (F_1,45_ = 28.45, *p* < 0.001), indicating a partial recovery of male fertility. Calculating the daily average fertility values for all four males revealed fluctuations in average fertility values during the first 15 d, followed by a drastic decline 1 wk prior to the onset of sterility. Furthermore, examination of the poststerility stage revealed a gradual increase in fertility until reaching a fluctuating pattern similar to the pre-sterility period (Figure 1A).

**Table 1:** Treatment groups, doses of busulfan used, and the fertility averages of the eggs laid throughout the experiment.

	Method of injection	Busulfan dosage, (mg/kg)	Fertility during presterility stage, (%)	Fertility during sterility window, (%)	Fertility during poststerility stage, (%)	Statistical differences between the pre-sterility stage and the poststerility stage
Quail	Intraperitoneal	40	58 ± 0.23	0	28 ± 0.15	F1,45 = 28.45, *p* < 0.001
	Intratesticular	12	70 ± 0.22	0	52.2 ± 0.23	F1,27 = 4.48, *p* = 0.044
	Intratesticular	20	71.4 ± 0.29	0	65 ± 0.24	F1,25 = 0.343, *p* = 0.563
Chicken	Intratesticular	20	44.8 ± 0.23	0	28.9 ± 0.09	F 1,40 = 7.003, *p* = 0.012

Subsequently, we sought to investigate the impact of busulfan intraperitoneal injections on the viability and fertility of chicken roosters (n = 4). However, a single intraperitoneal injection of 40 mg/kg BW resulted in 100% mortality in roosters within 7 d of the injection, as opposed to our findings in quails.

### The Effect of Busulfan Intratesticular Injections on Viability and Fertility in Quails and Roosters

To overcome the lethal consequences associated with intraperitoneal injection of busulfan in roosters and potentially improve the protocol for inducing male sterility in quails, we employed a different injection technique: direct busulfan injection into the testicles.

To determine the optimal concentration of busulfan for suppression of spermatogenesis with a minimal impact on male quail function, we administered busulfan at concentrations of 6, 12, and 20 mg/kg BW directly into both testicles; all three concentrations had no discernible effect on male viability. In terms of male sterility, the injection of 6 mg/kg BW into male quails (n = 2) resulted in a marginal and almost negligible impact on sterility. We observed a gradual decline in fertility starting from day 19 after the injection, reaching 0% at d 25 postinjection, followed by an immediate increase in sterility on d 26 (Figure 1B). On the contrary, after administering the 12 mg/kg BW dosage, quails (n = 2) exhibited a notably different response compared to the 6 mg/kg BW dose, with a complete loss of fertility occurring at d 18 after the injection. This sterility window persisted for a prolonged duration of 11 ± 5.66 d (Figure 1C). Lastly, in the 20 mg/kg BW dosage group (n = 2), a sterility window of 16.5 ± 2.12 d was observed, starting on d 19 after the injection and extending until d 35 (Figure 1D). Upon thoroughly examining these findings, it is evident that both the 12 mg/kg and 20 mg/kg dosages resulted in an average fertility of 70% ± 0.22 and 71.4% ± 0.29, respectively, during the presterility stage. However, in the post-sterility stage, the fertility averages declined to 52.2% ± 0.23 and 65% ± 0.24 for the 12 mg/kg and 20 mg/kg dosages, respectively (Table 1). These findings indicate that the 12 mg/kg BW busulfan dose administered directly into the testicles yields a significant decrease in male fertility subsequent to the sterility stage (F_1,27_ = 4.48, *p* = 0.044). Conversely, the administration of 20 mg/kg BW busulfan did not lead to a statistically significant variance (F_1,25_ = 0.343, *p* = 0.563) in the average fertility values of the pre- and post-sterility phases.

In light of the obtained findings, we determined that the 20 mg/kg BW busulfan dose is optimal for the intratesticular injections in quail. Next, we injected 20 mg/kg BW busulfan into a group of roosters (n = 4) intratesticularly. Our results revealed that administering the specified dosage to a rooster resulted in a sterility window lasting 27.2 ± 14.14 d (Figure 1E). The analysis of this dataset revealed notable fluctuations in the sterility of the males immediately following the injection, with a noticeable decline observed 1 wk prior to entering the sterility phase. Ultimately, the fertility average value reached 0% at 23.75 ± 2.9 d postinjection. In the pre-sterility period, approximately 44.8% ± 0.23 of the eggs were fertilized. Conversely, during the post-sterility stage, a significant decrease in fertility was observed compared to the pre-sterility stage (F _1,40_ = 7.003, *p* = 0.012), resulting in only 28.9% ± 0.09 fertile eggs (Table 1).

### Time-Dependent Histological Analysis of Spermatogenesis Following Intraperitoneal Busulfan Treatment in Quail

To study the temporal impact of busulfan on spermatogenesis, a busulfan dosage of 40 mg/kg BW was administered via an intraperitoneal injection into a cohort of 6 quails. Subsequently, a pair of quails were randomly selected and euthanized for analysis at specific intervals of 5, 10, and 15 d postinjection.

**Figure 2:**
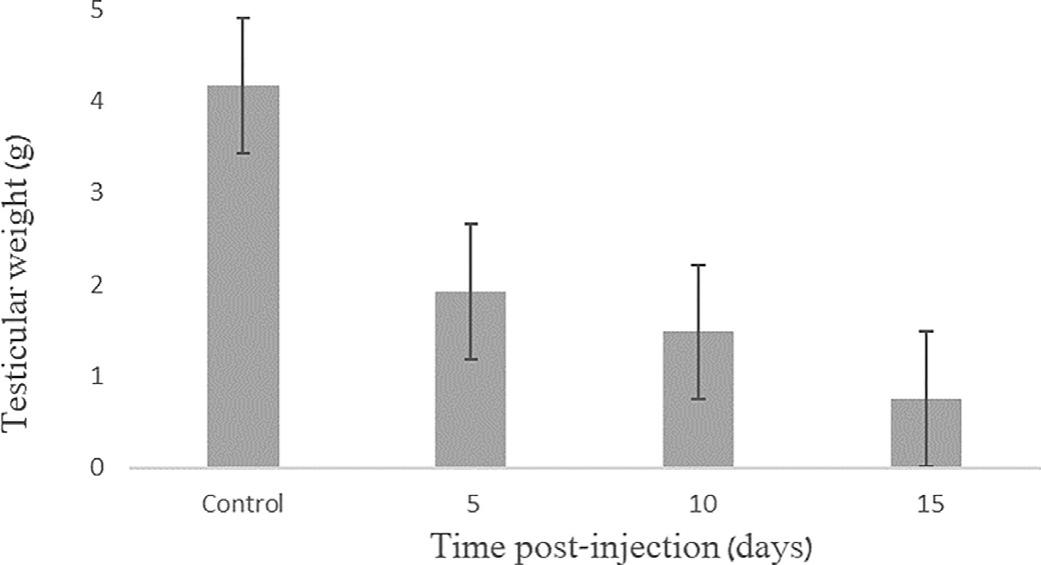
Effect of intraperitoneal busulfan treatment on the testicular weight. Quails received a single injection of 40 mg/kg BW.

To examine the morphological transformations following injection, we conducted a testicular weight measurement for each quail. The vehicle-only injected control quails displayed an average testicular weight of 4.1625 grams, whereas all busulfan-injected quails exhibited a decline in testicular weight (Table 2). Notably, a significant reduction in testicular weight was observed early on, starting on d 5 after injections, with an average weight of 1.9175 grams. Subsequently, a mild reduction in testicular weight persisted at d 10 and 15 postinjection compared to the 5-d mark, registering at 1.48 grams and 0.7525 grams, respectively. Importantly, these reductions were significantly smaller than those observed in the control group (F _3,4_ = 28.19, *p* = 0.004) (Figure 2).

**Table 2:** Documentation of the body and testes weight in quails, with or without busulfan treatment.

Group	Average body weight, (grams)	Average testes weight, (grams)	Gonado-somatic index, (%)
Control	225.235	4.1625	1.848
5 d after injection	163.035	1.9175	1.176
10 d after injection	222.64	1.48	0.665
15 d after injection	206.37	0.7525	0.365

**Figure 3:**
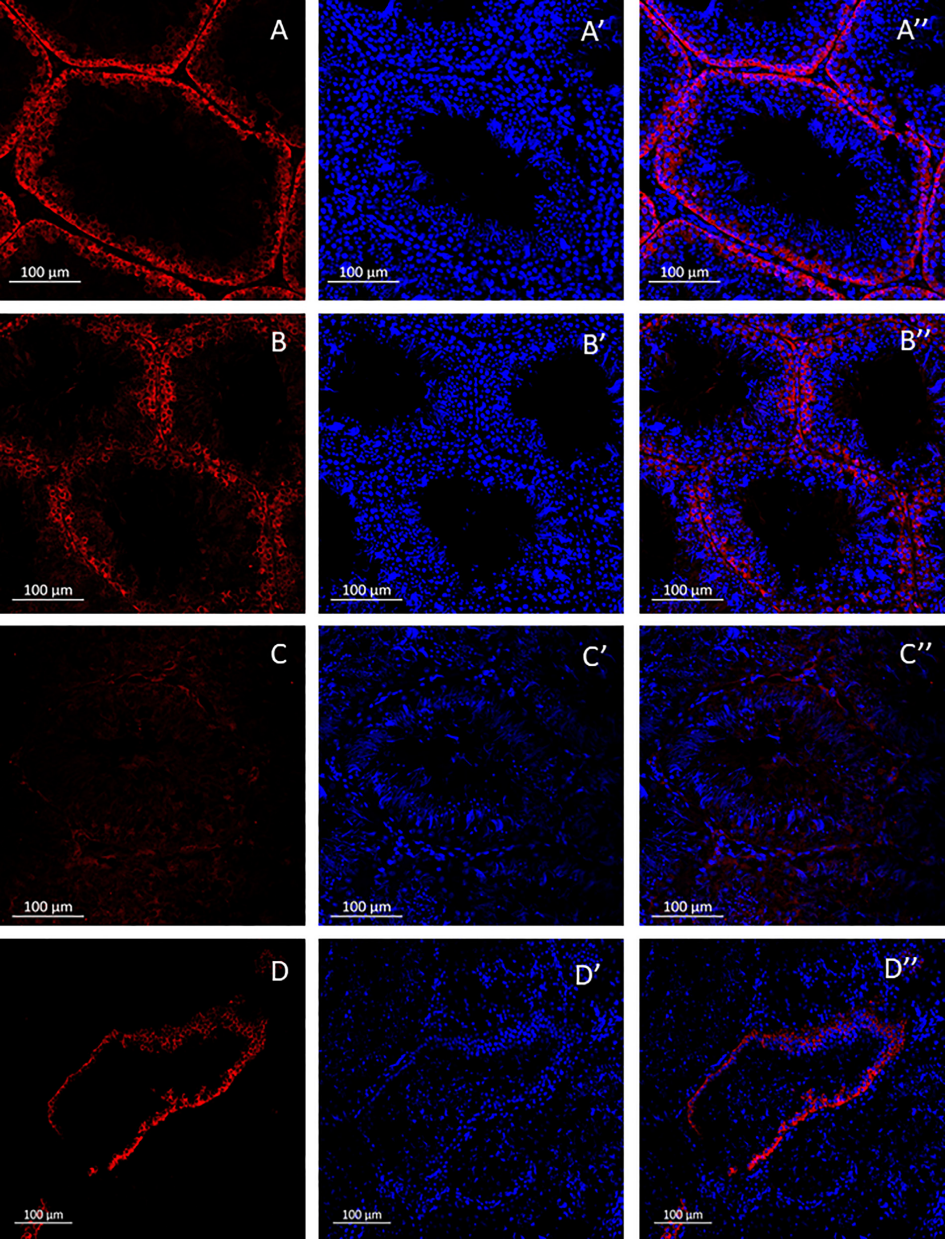
Temporal changes in quail testis morphology and SSCs after intraperitoneal injections of busulfan at 40 mg/kg. A, B, C, D - Hoechst staining (DNA) (blue). A', B', C', D' - localization of DAZL, a marker for SSCs (red). A'', B'', C'', D'' - merged images. A-A'' – control; B-B'' - 5 d after busulfan injection; C-C'' - 10 d after busulfan injection; D-D'' - 15 d after busulfan injection.

**Figure 4:**
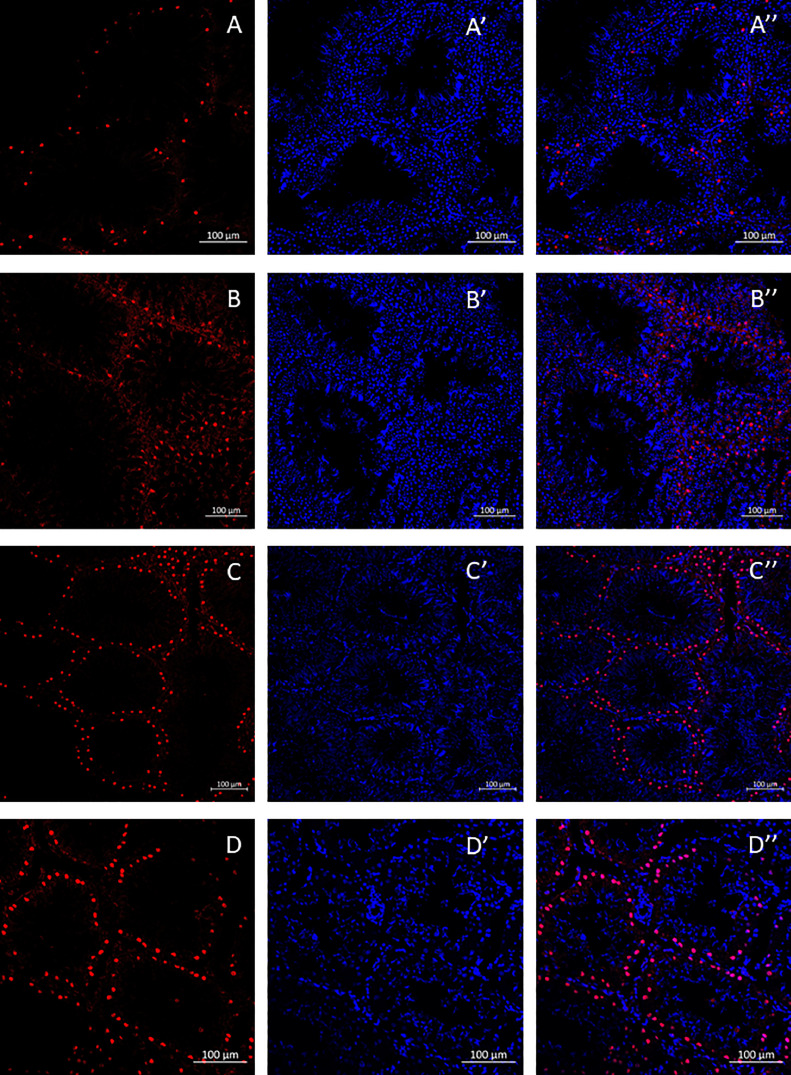
Temporal changes in quail testis morphology and Sertoli cells after intraperitoneal injection of 40 mg/kg BW busulfan. A, B, C, D - Hoechst staining (DNA) (blue). A', B', C', D' - localization of SOX9, a marker for SCs (red). A'', B'', C'', D'' - merged pictures. A-A'' – control; B-B'' - 5 d after busulfan injection; C-C'' - 10 d after busulfan injection; D-D'' - 15 d after busulfan injection.

To investigate the temporal dynamics of spermatogenesis associated with busulfan injection and the accompanying modifications in testicular morphology, the testicles were dissected and subjected to immunohistochemical processing, allowing the visualization and analysis of the cellular and molecular changes occurring within the testicular tissue over time. To identify SSCs, anti-DAZL antibodies were utilized, and the corresponding labeling was observed (Figure 3). Additionally, Sertoli cells (**SCs**) were labeled using anti-Sox9 antibodies (Figure 4). These immunohistochemical markers enabled the precise identification and localization of SSCs and SCs within the testicular tissue.

Regarding SSCs, notable changes were observed in the testicular tissue at various time points following the busulfan injection. At the 5-day mark (Figure 3B-B’’), a slight reduction in the diameter of seminiferous tubules was evident (268.6 ± 36.67µm) compared to the control group (305.5 ± 27.4µm) (Figure 3A-A’’); however, this reduction did not reach statistical significance (*p* = 0.214). In contrast, at the 10-d time point (Figure 3C-C’’), there was a nearly complete absence of SSCs, and the seminiferous tubules had a significantly lower average diameter of 160.22 ± 9.65 µm (*p* < 0.001) compared to the control. Following 15 d (Figure 3D-D’’), the tubules began to exhibit early signs of structural recovery and an increase in SSC number, indicating a partial restoration of their population; however, the tubules lacked the characteristic healthy, circular appearance, exhibiting noticeable abnormalities. Moreover, the seminiferous tubule diameter (146.4 ± 32.75µm) was significantly narrower compared to the control (*p* < 0.001).

Unlike SSCs, SCs displayed a higher degree of resilience to the busulfan injection, as minimal changes in the number of stained cells and morphology were observed throughout the entire experimental duration. Apart from a slight decrease in staining intensity observed on day 5 (Figure 4B-B’’), the overall impact on SCs was negligible (Figures 4A-A’’, 4C-C’’, 4D-D’’). This suggests that SCs exhibit strong resistance to the detrimental effects of busulfan, thus maintaining their functional integrity throughout the experimental timeline.

## DISCUSSION

### Intratesticular Injection of Busulfan is Not Toxic to Chicken and Quail

In this study, we found that a single dose of 40 mg/kg BW administered intraperitoneally had no adverse effects on quail viability but led to the suppression of male fertility, in accordance with previously published research. In contrast, the same dose exhibited considerable toxicity in roosters, ultimately leading to 100% mortality. Earlier research conducted in chickens also had some outcome variability; for example, a single intraperitoneal injection of 35 mg/kg or 60 mg/kg BW resulted in the deaths of two out of seven roosters (Yu et al., 2010) or all roosters (Tagirov & Golovan, 2012), respectively. Nevertheless, 60 mg/kg BW of busulfan, when administered intraperitoneally in two split doses of 40 mg/kg and 20 mg/kg, did not affect roosters’ viability (Ghadimi et al., 2017; Tagirov & Golovan, 2012). The discrepancies between the current study and the work done by Tagirov & Golovan, 2012 may be attributed to the differences in roosters’ age (mature vs. pubertal, respectively) or breed (Lohmann vs. White Leghorn, respectively). Additionally, while our study and the study by Ghadimi et al., 2017 involved injections into mature roosters, distinct breeds were used (Lohmann vs. Ross 308, respectively).

To the best of our knowledge, all earlier reports involving busulfan in chickens and quails employed exclusively intraperitoneal injections. For the first time, we utilized a different strategy by administering busulfan intratesticularly to quails and chickens in our study. In quails, the effect of intratesticular busulfan injection was similar to that of intraperitoneal injection, as it had no negative impact on male viability yet induced a temporary sterility window, followed by a successive recovery of fertility. Notably, in roosters, intratesticular injection of busulfan significantly improved vitality compared to intraperitoneal injection, indicating that intratesticular injection is superior to the previously employed intraperitoneal injections.

### A Higher Dose of Intratesticular Injection of Busulfan is Required to Suppress Spermatogenesis in Quails Compared to Mammals

Our results showed that a high dose of intratesticular injection of busulfan is required to inhibit spermatogenesis in quail. We found that while the intratesticular injection of 6 mg/kg and 12 mg/kg BW of busulfan had only a marginal effect on male fertility, the injection of 20 mg/kg BW induced male sterility, similar to the effect of intraperitoneal busulfan injection of 40 mg/kg BW. In contrast to other animal models where the recommended doses for intratesticular injection range between 2 and 5 mg/kg BW (Lin et al., 2017; Mobarak et al., 2022; Qin et al., 2016a,b; Singh et al., 2022), we found that in quails the most efficient dose to suppress spermatogenesis is 20 mg/kg BW, a much higher dosage compared to mammals.

A deeper exploration is required to understand the need for high doses of busulfan intratesticular injection to suppress spermatogenesis in quail. One possible explanation can be that quail SSCs are more resistant to busulfan than mammalian SSCs, supported by the faster recovery of fertility in quail compared to mammals. In quails, fertility restoration occurs as early as 41.3 ± 3.5 d after the busulfan intraperitoneal injection. On the contrary, in mice, the effect of busulfan on male fertility is stronger, as the male fertility recovery was observed after a longer period (for example, 110 d (Gutierrez et al., 2016) or 26 to 30 wk (Zohni et al., 2012), depending on the mouse strain) post-injection.

### Characterization of the Time-Dependent Effect of Busulfan on Quail Spermatogenesis

Understanding the time-dependent effect of busulfan on quail spermatogenesis is essential for establishing the best time for genetically modified SSC transplantation to generate transgenic animals. However, the temporal effect of busulfan on avian spermatogenesis has not been studied extensively. The endpoint effect of busulfan injection on chicken spermatogenesis using histological analysis was described previously, showing complete suppression of spermatogenesis (Tagirov & Golovan, 2012) or no spermatogenesis activity in the seminiferous tubules (Ghadimi et al., 2017). In the current study, we comprehensively characterized the temporal changes in quail spermatogenesis following the intraperitoneal injection of a single 40 mg/kg BW busulfan dose.

Our findings showed that 5 d after injection, there were no significant changes in testicular weight or spermatogenesis features (i.e., the diameter of seminiferous tubules and SSC count). Ten d after injection, a drastic effect of male spermatogenesis was evident, reflected by a significant reduction in testis weight, the disappearance of SSCs, and abnormalities in seminiferous tubules. At day 15 postinjection, spermatogenesis appeared to recover as SSCs could be detected again, although the testis weight was significantly lower compared to the control group. On the other hand, fertility tests in the injected quails indicated a complete loss of fertility occurring at 20 ± 1.73 d postinjection and fertility restoration after an average of 41.3 ± 3.5 d postinjection. Thus, the histological examination revealed that a complete loss of spermatogenesis occurs 10 d after injection, and the first signs of recovery appear 15 d after injection, while male fertility is fully restored only after 25 d on average. This finding holds particular significance as it suggests that successful transplantation of PGCs can be performed optimally within the critical window spanning 10 to 15 d after the intraperitoneal busulfan injection.

In conclusion, transgenic birds serve as valuable research platforms with established methods for their generation. Intriguingly, manipulating spermatogonial stem cells (SSCs) in recipient testes has shown promise in enhancing transgenesis efficiency. In mammals, the use of busulfan, particularly through intratesticular injection, offers a convenient means of depleting endogenous SSCs, thereby facilitating the successful transplantation of genetically modified cells. For the first time, our study demonstrates the efficacy of intratesticular busulfan injection in inducing male sterility without adverse effects on viability in quails and chickens. These findings provide critical insights for refining protocols and improving transgenesis efficiency in avian models.

## DISCLOSURES

The authors declare no conflicts of interest.
